# Jellyfish Life Stages Shape Associated Microbial Communities, While a Core Microbiome Is Maintained Across All

**DOI:** 10.3389/fmicb.2018.01534

**Published:** 2018-07-12

**Authors:** Michael D. Lee, Joshua D. Kling, Rubén Araya, Janja Ceh

**Affiliations:** ^1^Department of Biological Sciences, University of Southern California, Los Angeles, CA, United States; ^2^Instituto de Ciencias Naturales Alexander von Humboldt, Universidad de Antofagasta, Antofagasta, Chile; ^3^Laboratory of Microbial Complexity and Functional Ecology, Institute of Antofagasta, University of Antofagasta, Antofagasta, Chile; ^4^Centre for Biotechnology and Bioengineering, Universidad de Chile, Santiago, Chile

**Keywords:** jellyfish microbiome, *Chrysaora plocamia*, jellyfish-bacteria, polyp stages, Humboldt Current System

## Abstract

The key to 650 million years of evolutionary success in jellyfish is adaptability: with alternating benthic and pelagic generations, sexual and asexual reproductive modes, multitudes of body forms and a cosmopolitan distribution, jellyfish are likely to have established a plenitude of microbial associations. Here we explored bacterial assemblages in the scyphozoan jellyfish *Chrysaora plocamia* (Lesson 1832). Life stages involved in propagation through cyst formation, i.e., the mother polyp, its dormant cysts (podocysts), and polyps recently excysted (excysts) from podocysts – were investigated. Associated bacterial assemblages were assessed using MiSeq Illumina paired-end tag sequencing of the V1V2 region of the 16S rRNA gene. A microbial core-community was identified as present through all investigated life stages, including bacteria with closest relatives known to be key drivers of carbon, nitrogen, phosphorus, and sulfur cycling. Moreover, the fact that half of *C. plocamia*’s core bacteria were also present in life stages of the jellyfish *Aurelia aurita*, suggests that this bacterial community might represent an intrinsic characteristic of scyphozoan jellyfish, contributing to their evolutionary success.

## Introduction

Animals are becoming increasingly appreciated and studied as metaorganisms, or supraorganismal structures, closely associated with multi-lineage consortia of microorganisms (i.e., bacteria, archaea, eukaryotes, and viruses), together representing a dynamic assemblage that results in a co-metabolism between the animal and its microbiome (McFall-Ngai et al., 2013). These relationships have evolved so closely that in some cases host development depends on signals from these microbes, and its immune system may even recognize specific microbes as part of itself (Gilbert et al., 2012; McFall-Ngai et al., 2013). As bacterial life had already existed for about three billion years when animals first evolved (Knoll, 2015), microbe-animal interactions are likely as old as animals themselves (Bosch, 2013). Based on their early appearance on the evolutionary scene, cnidarians were likely amongst the first animals to establish associations with microorganisms.

With the four groups Anthozoa, Cubozoa, Hydrozoa, and Scyphozoa, the phylum is comprised of diverse taxa that inhabit all parts of the world’s ocean. A simple anatomy made up of two germ layers, separated by an extracellular matrix, and highly complex stinging cells (cnidocytes) characterizes these basal animals (Daly et al., 2007; Fautin, 2009; Rogers, 2009).

Within the cnidarians, corals and scyphozoans stand out as particularly important taxa: reef-building corals are important ecosystem engineers of high ecological and economic value, while scyphozoans are infamous for their negative effect on marine ecosystems, fisheries and industries (Richardson et al., 2009; Graham et al., 2014).

A staggering level of developmental plasticity (Ceh et al., 2015) and the ability to respond to spatiotemporal environmental fluctuations quickly allows scyphozoans to exploit a vast variety of habitats and resources (Thein et al., 2013). Sexual reproduction in pelagic medusae results in a motile planula that settles in the benthos, metamorphosizes, and grows into a polyp. Such polyps reproduce asexually, multiplying in numbers and producing new medusae when environmental factors are appropriate. While most jellyfish-bloom studies have focused on the more conspicuous medusae, which appear seasonally in ocean surface waters, little is known about their polyp counterparts. However, relative longevity coupled with the potential to multiply in large numbers, and their capacity to withstand a wide range of adverse environmental conditions, make the polyp a vital part of the scyphozoan life cycle. In fact, the capacity of scyphozoans to proliferate has been argued to reside in the sessile polyps (Arai, 2009).

Depending on the species, polyps can proliferate asexually in various ways (reviewed in Adler and Jarms, 2009), including cyst formation (Vagelli, 2007; Lucas et al., 2012), where polyps in the orders Rhizostomae (suborder Dactyliophorae) and Semaeostomae (Arai, 2009) form cysts against substrates by pedal disks or stolons. These so called podocysts are chitin-enclosed, contain stored reserves of organic compounds (Arai, 2009), and can stay dormant for long periods of time (Black, 1981; Thein et al., 2012). When appropriate environmental conditions occur, a small polyp excysts and develops into a fully functional polyp, capable of producing more podocysts and medusae (Arai, 2009). The strategy of cyst formation can contribute to an increase in polyp numbers, as well as to the animal’s survival, since podocysts are resilient to environmental conditions that polyps do not survive, e.g., severe hypoxia (Ishii et al., 2008; Levin et al., 2009; Thein et al., 2012) or predation by nudibranchs (Cargo and Schultz, 1967).

Being amongst the first and simplest animals existing, cnidarians are an important group to investigate regarding the evolution of animal-microbial symbioses, and while bacterial partners have been well studied in other cnidarians (Rosenberg et al., 2007), investigating jellyfish-microbial interactions has only begun more recently (Weiland-Bräuer et al., 2015; Cleary et al., 2016; Daley et al., 2016; Viver et al., 2017). Associated microorganisms can provide a vast genetic repertoire to a host, expanding its metabolic potential and adaptability to environmental changes (Lapierre and Gogarten, 2009; McFall-Ngai et al., 2013). Considering the complexity of the jellyfish life cycle and the large variety of biological processes associated with it (e.g., different phenotypes, reproductive strategies, ecosystems, and environmental conditions), jellyfish could be expected to benefit from a versatile microbiome that may change in accordance with the current needs of the animal – or perhaps more aptly considered as a change in accordance with the current needs of the supraorganismal structure.

The large and abundant jellyfish *Chrysaora plocamia* (Lesson 1832) occurs along the Atlantic coast of South America, as well as in the Chilean-Peruvian Humboldt Current System where it has been reported for its negative effects on anchovy fisheries and tourism (Quiñones et al., 2013). Its potential to severely alter the highly productive Humboldt Current System (Thiel et al., 2007) makes it a key species to study (Riascos et al., 2013; Riascos et al., 2015). As in other *Chrysaora* species, e.g. in *Chrysaora pacifica* (Thein et al., 2013), cyst formation is the only known mode for polyp propagation in *C. plocamia*, and therefore bears a particularly important role for the proliferation and survival of this large and abundant jellyfish.

Here, in an attempt to shed more light on bacteria associated with jellyfish, and how such associations may be involved in the important process of polyp proliferation via cyst formation, we assessed microbial communities harbored by three life stages – polyps, podocysts, and excysts – in the jellyfish *C. plocamia* using MiSeq Illumina paired-end tag sequencing of the V1V2 region of the 16S rRNA gene.

## Materials and Methods

### Organism Collection and Maintenance

Polyp cultures were established from *C. plocamia* medusae collected at different times and in different places in Northern Chile: in December 2014 and January 2015 in Bolsico (23°27″18′S, 70°36″08′W); in February 2015 in Bahia Inglesa (27°06″44′S, 70°54″52′W); and in April 2015 in Mejillones Bay (23°04″23′S, 70°24″50′W). Medusae were collected by scuba divers from a maximum depth of 15 m, caught with a dip-net, individually placed into plastic bags, stored in a cooler, and transported to the laboratory for further processing. Oral arms were inspected for planulae, sectioned, and placed onto a mesh (1 mm mesh-size) in a 10 L plastic box in 1 μm filtered seawater. To initiate shedding of planulae the container was slowly moved and the accumulated planulae were harvested from the bottom (Riascos et al., 2013; Ceh and Riascos, 2017). Two hundred planulae from individual medusae were placed into individual plastic receptacles, containing 250 mL of 1 μm filtered seawater and a settlement structure, i.e., a plastic petri dish, placed upside-down on the water surface for planulae to settle on the underside. Once most planulae had settled, settlement substrates were transferred into an 800 L flow-through tank fed by ambient seawater and kept at a day night rhythm of 12:12 h. The water temperature was not manipulated and neither were animals artificially fed at any time in order to assure the most natural food conditions possible. Polyps grew to a 16-tentacle-stage within 8 weeks and shortly after produced podocysts, often followed by strobilation.

### Sampling Procedure

**FIGURE 1:**
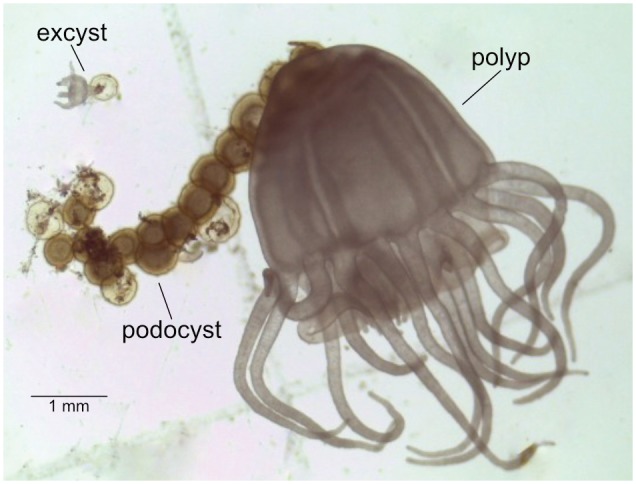
*Chrysaora plocamia* mother-polyp with podocysts and excyst.

Mother polyps, podocysts, and excysts (**Figure 1**) were sampled from each cohort. To test whether the nearby spatial presence of a mother polyp may influence the microbial composition of podocysts, two groups of podocysts were distinguished: the first included podocysts near the mother polyp; while the second group of podocysts included those that were physically isolated from any polyp and originated from a different mother polyp. Altogether this resulted in the following four groups being investigated: mother polyps (1), their podocysts (2) and excysts (3); and then isolated podocysts (4). One polyp, six to eight podocysts (∼2 weeks old) and one excyst (4 tentacle stage, ∼2 days old) per cohort were harvested. For replication, we opted to cover multiple time-points with individual samples per time-point, rather than multiple samples from only one time-point. Therefore, there were four replicates of each life stage, each collected from a different cohort (time), and it is these groups of 4 that were used for statistical analysis.

Only non-feeding polyps and excysts were sampled. Using a stereomicroscope, all life stages were carefully scraped off the settlement substrates with a sterile micropipette tip, washed in artificial seawater to remove non-associated bacteria and salt, placed into 2 mL cryo-valves, snap-frozen in liquid nitrogen, and stored in -80°C until further processing. Podocysts were dipped into ethanol before the washing step to eliminate bacteria on the outside of the capsule.

### Nucleic Acid Extractions

For the isolation of bacterial genomic DNA a chelex extraction was performed using Instagene Matrix (Bio-Rad Laboratories, Hercules, CA, United States), following the protocol described in Montero-Pau et al. (2008). Samples were defrosted, transferred into individual 2 mL tubes containing 20 μL of alkaline lysis buffer, and crushed against the side of the tube with a sterile pipette tip. Subsequently 100 μL of Instagene Matrix were added, and samples were incubated for 30 min at 60°C and for 8 min at 98°C. Finally, the samples were centrifuged at 10,000 rpm for 5 min, and the supernatant containing the DNA was stored at -20°C until needed.

### DNA Tag Sequencing and Processing

DNA extracted from the 16 samples was sequenced by a commercial vender (Molecular Research LP, MR DNA, Shallowater, TX, United States). Illumina MiSeq (2 × 300 bp) paired-end tag sequencing was performed with primers targeting the V1V2 region of the 16S rRNA gene (27f: 5′-AGRGTTTGATCMTGGCTCAG-3′; 519r: 5′-GTNTTACNGCGGCKGCTG-3′). Library preparation and sequencing was carried out at the facility following the Illumina MiSeq DNA library preparation protocol. Tag data curation and processing were performed within mothur v.1.36.1 (Schloss et al., 2009), following the *mothur* Illumina MiSeq Standard Operating Procedure (Kozich et al., 2013). Briefly, forward and reverse reads were merged, any sequences with ambiguous base calls or homopolymers longer than 8 bp were removed, and primers were trimmed allowing for 0 mismatches. These sequences were then aligned to the mothur-recreated Silva SEED database v123 (Yarza et al., 2008). Sequences were then preclustered at a near 1% dissimilarity, as this has been shown to minimize the representation of spurious sequences due to sequencing error (Kozich et al., 2013). Chimeric sequences were screened with UCHIME in de novo mode (Edgar et al., 2011). Remaining sequences were clustered into Operational Taxonomic Units (OTUs) at 3% or less dissimilarity via the average neighbor method, and representative sequences for each OTU were generated using the abundance method. The resulting OTU count matrix was filtered with a conservative cutoff threshold of 0.005% of total reads for each individual OTU. This criterion has been recommended when a mock community is not incorporated (Bokulich et al., 2013), however, precludes any richness estimators that utilize singletons in their calculations. Taxonomy was called using the Ribosomal Database Project (RDP) online classifier (Cole et al., 2013). It should be remembered that due to the variability regarding 16S rRNA gene copy number in different organisms all relative abundances discussed herein are in the context of sequences recovered, rather than in relative organismal abundance.

### OTU Count Matrix Visualizations, Statistics, and Differential Abundance Analysis

Our filtered OTU count matrix was imported into *RStudio* v0.98.1091 (Racine, 2012) and primarily analyzed with the packages vegan v2.3-0 (Oksanen, 2015), phyloseq v1.16.2 (McMurdie and Holmes, 2013), and *DESeq2* v1.12.4 (Love et al., 2014) with default function settings unless otherwise noted. Using Wisconsin-transformed values of our filtered input count table (Supplementary Table S1), a Bray–Curtis dissimilarity matrix was generated and used as input for hierarchical clustering with *hclust*(method = ward.D2), principle coordinates analysis (PCoA) performed with *betadisper*() to also assess within group variation, permutational ANOVA performed with *adonis(*permutations = 99999), and *meandist*() to assess mean within- and between-group dissimilarities. The filtered, non-transformed count matrix and RDP taxonomy were used to create a *phyloseq* object, which was then filtered to include only OTUs with greater than five reads. This *phyloseq*-filtered count matrix was used to generate all relative abundance values and taxonomic profiles, and as input into *DESeq2* for differential abundance analysis. *DESeq*() was called with default parameters, and when filtering contrast tables an adjusted *p*-value cutoff of 0.01 was used for differences in relative abundances to be considered statistically significant.

### Identification of Consistently Present OTUs Across All *C. plocamia* Samples

All OTUs not identified as significantly differentially abundant in any of the six possible contrasts between our four groups were further screened to find those consistently present in all groups. Of these, we further filtered to include only those OTUs that were present in each of the 16 samples, and identified this as the bacterial ‘core’ in our *C. plocamia* data.

### Incorporation of *Aurelia aurita* Bacterial Community Data

Raw sequence data from a recently published marker-gene study focusing on bacterial communities associated with the scyphozoan jellyfish *Aurelia aurita* (Weiland-Bräuer et al., 2015) were downloaded from NCBI’s Sequence Read Archive (SRA). While both jellyfish species belong to the order Semaeostomeae, *A. aurita* is of the family Ulmaridae and *C. plocamia* belongs to the Pelagiidae. The Weiland-Bräuer work utilized different primers than the current study, but targeted the same V1V2 region of the 16S rRNA gene. We processed these data within *mothur* in the same manner as described above to generate representative OTU sequences from that study, then performed a BLASTN (Altschul et al., 1990) search of our ‘core’ identified bacterial sequences against these to find those OTUs that were similar (>98.5% ID over >90% of the length of the query). As Weiland-Bräuer et al. (2015) included surrounding seawater samples in their study, we also used these data to remove OTUs that were present in any quantity in water samples. This left a subset of OTUs found to be present in both our defined ‘core’ microbiome of *C. plocamia* as well as within *A. aurita* samples.

### Accession Information

The raw Illumina MiSeq sequence data generated herein have been uploaded to NCBI’s Sequence Read Archive (SRA) under BioProject accession number PRJNA413177.

## Results

### Overview of the *C. plocamia* Bacterial Microbiome

**FIGURE 2:**
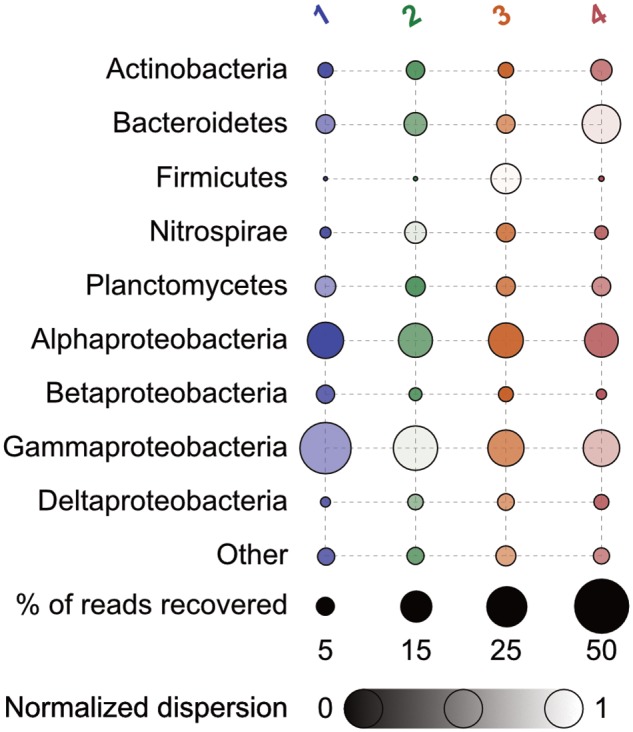
Percent relative abundance of 16S rRNA genes recovered of major taxa by group. Bubbles are faded according to the (log + 1)-transformed variance within each normalized across all to a scale of 0 to 1 – those with less variance appear darker. Numbers correspond as follows: 1, polyps; 2, podocysts; 3, excysts; and 4, isolated podocysts. *N* = 4 for each group.

At a broad taxonomic level, bacterial communities across the group types investigated (polyps, two groups of podocysts, and excysts), were dominated by Gammaproteobacteria, Alphaproteobacteria, Bacteroidetes, and Planctomycetes, together comprising ∼72.4 ± 13.5% (mean ± 1 standard deviation) of the total sequences recovered in each group (**Figure 2**). No statistically significant differences were found in relative abundance of recovered sequences between groups at this coarse level of taxonomic resolution, except for the class Betaproteobacteria, that was significantly more abundant in the polyp group as compared to the isolated podocyst group (∼5.7 ± 2.5% compared to ∼1.9 ± 0.7%, Tukey HSD *p* = 0.02; Supplementary Figure S1). The Gammaproteobacteria class was proportionally the most abundant major taxon as represented by the recovered sequences – comprising ∼32 ± 17% in each sample. This taxon’s relative abundance was driven primarily by members of the orders Alteromonadales (primarily *Shewanella* spp.), Legionellales (*Coxiella* spp.), and Methylococcales (*Methylobacter* spp.). Alphaproteobacteria was the next most dominant major taxon making up ∼21.4 ± 4% of recovered sequences with the majority of which derived from OTUs belonging to the genus *Bradyrhizobium* within the *Rhizobiales*. Members of the family Flavobacteriaceae were the most prominent within the Bacteroidetes, and the genera *Blastopirellula*, *Pirellula*, and *Rhodopirellula* were the dominant representatives recovered from the Planctomycetes. Within the Betaproteobacteria that accounted for significant differences in relative abundance between polyps and isolated cysts, *Ralstonia* and *Cupriavidus* within the Burkholderiales were most prominent.

### Bacterial Community Heterogeneity Between Polyps and Cysts

**FIGURE 3:**
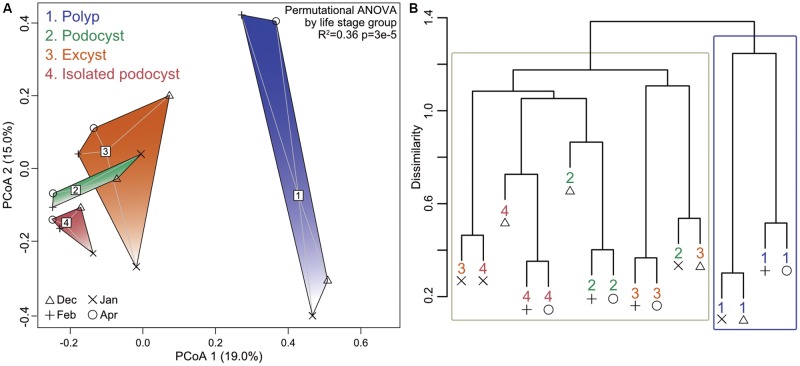
Principal coordinates analysis ordination **(A)** and hierarchical clustering **(B)** showing separation of groups based on Bray–Curtis dissimilarities. Group labels and symbols for collection dates indicated in **(A)** apply to both panels. Blue box in **(B)** surrounds all four polyp samples while the tan box surrounds all other sample types. The permutational ANOVA resultant *p*-value is listed in **(A)**.

Permutational ANOVA revealed that neither date nor location of medusa sampling were predictive of community structure to any significance (*p* = 0.4), while the four life-stage groupings were able to account for 36% of the observed variance (*p* = 3e-5). Principal coordinates analysis and hierarchical clustering both demonstrated some overlap among cyst groups, but a strong separation of polyp samples from all cyst samples (**Figure 3**). Comparing the means of within- and between-group dissimilarities further supported this distinction as all cyst groups were found to be more similar to each other than any polyp-to-cyst contrast (Welch *t*-test, *p* = 0.008; **Table 1**, Supplementary Table S2 and Supplementary Figure S2). Taken together, these results demonstrate that polyp bacterial communities are significantly different from their podocyst and excyst counterparts.

**Table 1:** Between group Bray–Curtis dissimilarities and differentially abundant OTUs comparing all polyp-to-cyst group contrasts with all cyst-to-cyst group contrasts.

	Between group dissimilarities			Differentially abundant OTUs								
Contrast	Mean diss.	Mean of means ± 1 SD	Welch *t*-test	Increased	Mean inc. ± 1 SD	Welch *t*-test	Decreased	Mean dec. ± 1 SD	Welch *t*-test	Total diff.	Mean diff. ± 1 SD	Welch *t*-test
1v2	0.922	0.914 ±0.01	*p* = 0.01	8	9 ±5.6	*p* = 0.15	25	28.3±3.06	*p* = 0.001	33	37.3±5.86	*p* = 0.003
1v3	0.903			4			31			35		
1v4	0.918			15			29			44		
2v3	0.752	0.762 ± 0.033		2	2 ± 2		3	4.33 ± 2.31		5	6.3 ± 4.16	
2v4	0.799			0			3			3		
3v4	0.735			4			7			11		

*Groups are identified by number: 1, polyps; 2, podocysts; 3, excysts; and 4, isolated podocysts.*

### Differential Abundance Analysis

**FIGURE 4:**
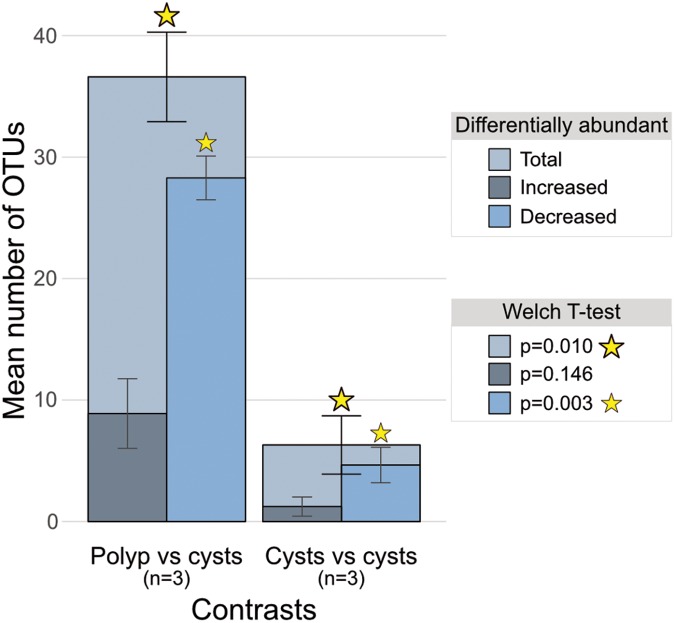
Bar charts of mean total differentially abundant OTUs overlain with means of those increased and those decreased in polyp-to-cyst group contrasts and cyst-to-cyst group contrasts. The 3 polyp-to-cyst contrasts included: Polyps–Podocysts; Polyps–Excysts; and Polyps–isolated podocysts. The 3 cyst-cyst contrasts included: Podocyst–Excyst; Podocyst–isolated podocyst; and Excyst–isolated podocyst. The means between the 3 polyp vs. all cyst groups contrasts and the 3 cyst vs. cyst contrasts were found to be significantly different via the Welch *t*-test (*p* = 0.01).

To ascertain which particular OTUs were causing the difference between polyp and cyst microbial communities, we employed a differential abundance analysis across our 4 groups resulting in 6 pairwise comparisons. This identified significantly more differentially abundant OTUs in all 3 polyp-to-cyst group contrasts than in any of the 3 cyst-to-cyst group contrasts (**Table 1** and **Figure 4**). Particularly of note, a significantly greater amount of those OTUs called as differentially abundant between any polyp-to-cyst contrast were lower in abundance in the polyp stage as compared to cysts (Welch *T*-test, *p* = 0.03). All differentially abundant OTUs for each of the 6 contrasts are presented in Supplementary Table S3.

*DESeq2*’s approach for identifying differential abundance between groups is dependent on the variances within the groups being contrasted. As all cyst groups were similar to each other, as presented above, we combined these 3 groups into one group of 12 samples and contrasted them with the polyp group of 4 samples in order to better assess which OTUs were causing the large dissimilarities between polyps and cysts. Contrasting these 2 groups revealed 7 OTUs that were relatively more abundant in the polyp stage as compared to 56 that were relatively more abundant in cyst stages (Supplementary Table S4). Those more abundant in cyst stages included chemolithoautotrophs (of the genera *Nitrospira*, *Nitrospina*, *Thiogranum*, and *Desulfovermiculus*), nitrogen fixers belonging to the order Rhizobiales, and many OTUs with closest relatives involved with nitrogen and sulfur reduction (Supplementary Table S4).

To examine community members that might be potentially integral to the dormant podocyst stage, we combined the two podocyst groups (nearby and isolated) and performed differential abundance analysis across these 3 groups resulting in 3 pairwise comparisons: polyps vs. podocysts, polyps vs. excysts, and excysts vs. podocysts (Supplementary Table S5). We further parsed these results to identify which OTUs had significantly increased relative abundances in the podocyst group as compared to the polyp group, but were not significantly more abundant in the excyst group as compared to the polyp group, revealing 26 OTUs (presented in Supplementary Table S6). Further, this approach elucidated 3 OTUs that were significantly more abundant in the excyst group as compared to the podocyst group (Supplementary Table S5, bottom table “Excysts vs. Podocysts”).

### A ‘Core’ Bacterial Microbiome

Thirty-seven of the 239 OTUs incorporated in the differential abundance analysis were present in all 16 samples (Supplementary Table S7) – comprising ∼23% of total reads recovered. Half of these had closest non-redundant BLAST hits to other marine microbiome studies (e.g., seaweed, sponges, sea squirts, and sea horses; highlighted in Supplementary Table S7), and 16 were found to be present in the *A. aurita* microbiome as well (Weiland-Bräuer et al., 2015; **Table 2**, with additional information in Supplementary Table S8). These included nitrogen cyclers, chemolithoautotrophs, methylotrophs, methane oxidizers, and even polycyclic aromatic hydrocarbon (PAH) degraders (**Table 2** and Supplementary Table S8).

**Table 2:** Bacterial OTUs present in all 16 of *C. plocamia* samples and also found in *A. aurita* (Weiland-Bräuer et al., 2015), along with potential functional niches based on best blast hits to characterized relatives.

Class	Order	Family	Genus species	Potential activities/enzymes	%ID	Accession	Otu ID
Alpha	Kordiimonadales	Kordiimonadaceae	*Emcibacter nanhaiensis*	Alkaline phosphatase^1^	91	NR_136430	Otu000185
	Rhizobiales	Bradyrhizobiaceae	*Afipia birgiae*	Nitrate reduction^2^	100	NR_025117	Otu000004
		Rhodobiaceae	*Afifella pfennigii*	Nitrogen fixation, sulfate reduction ^3,4^	90	NR_117117	Otu000011
	Rhodobacterales	Rhodobacteraceae	*Phaeobacter caeruleus*	Nitrate reduction, alkaline phosphatase^5^	99	NR_118542	Otu000051
	Rhodospirillales	Rhodospirillaceae	*Thalassospira tepidiphila*	Nitrate reduction, PAH degredation^6^	100	NR_041492	Otu000100
	Sphingomonadales	Sphingomonadaceae	*Sphingopyxis flavimaris*	Chemoorganotroph^7^	98	NR_025814	Otu000226
Beta	Nitrosomonadales	Methylophilaceae	*Methylobacillus glycogenes*	Methylotrophy^8^	93	NR_104760	Otu000039
		Nitrosomonadaceae	*Nitrosospira multiformis*	Chemolithoautotroph, ammonia oxidation, denitrification^9^	98	NR_074736	Otu000055
Gamma	Alteromonadales	Alteromonadaceae	*Marinobacter nitratireducens*	Nitrate reduction^10^	91	NR_136469	Otu000043
		Shewanellaceae	*Shewanella aestuarii*	Nitrate reduction, alkaline phosphatase^11^	97	NR_135728	Otu000001
	Chromatiales	Chromatiaceae	*Thiorhodococcus minor*	Sulfur oxidation, nitrogen fixation^12^	90	NR_116948	Otu000128
	Methylococcales	Methylococcaceae	*Methylobacter tundripaludum*	Methanotrophy, nitrogen fixation^13^	94	NR_042107	Otu000005
			*Methylobacter tundripaludum*	Methanotrophy, nitrogen fixation^13^	94	NR_042107	Otu000655
	Xanthomonadales	Xanthamonadaceae	*Stenotrophomonas pavanii*	Nitrogen fixation^14^	99	NR_118008	Otu000023
Delta	Desulfuromonadales	Desulfuromonadaceae	*Pelobacter carbinolicus*	Sulfur reduction^15^	79	NR_075013	Otu000008
Nitrospira	Nitrospirales	Nitrospiraceae	*Nitrospira moscoviensis*	Chemolithoautotroph, nitrite oxidation, ammonification^16^	84	NR_029287	Otu000015

*(^1^Liu et al., 2015; ^2^La Scola et al., 2002; ^3^Urdiain et al., 2008; ^4^Caumette et al., 2007; ^5^Vandecandelaere et al., 2009; ^6^Kodama et al., 2008; ^7^Yoon and Oh, 2005; ^8^Doronina et al., 2014; ^9^Norton et al., 2008; ^10^Vaidya et al., 2015; ^11^Park and Jeon, 2013; ^12^Imhoff, 2005; ^13^Wartiainen et al., 2006; ^14^Ramos et al., 2011; ^15^Aklujkar et al., 2012; ^16^Ehrich et al., 1995).*

## Discussion

The sophistication of scyphozoan jellyfish lies in their simplicity, versatility, and quite possibly in their microbiome. Here we discuss the microbiota identified in three life stages of the jellyfish *C. plocamia*, and reveal the microbial members found to be more abundant in specific developmental stages as well as the fraction of microorganisms more stably shared between all. Moreover, potentially intrinsic jellyfish-associated bacterial communities are described and discussed.

### Bacterial Communities Vary by Life Stage

At a coarse taxonomical level, bacterial communities across all investigated life stages were dominated by the same major taxa (Gammaproteobacteria, Alphaproteobacteria, Bacteroidetes, and Planctomycetes; **Figure 2**), with only the class Betaproteobacteria being significantly more abundant in the polyp stage as compared to the isolated podocysts (Supplementary Figure S1). However, analysis at the sequence-level (OTU-level) revealed groupings according to life stage (**Figure 3**), rather than by sampling location/time of medusae (and their respective colonizer pools). This type of life stage specificity has also been shown in the scyphozoan *A. aurita* where life stages involved in the polyp-to-medusa transformation (polyp, strobila, ephyra, and juvenile medusa) harbored distinct microbiota (Weiland-Bräuer et al., 2015). Such results are not surprising when considering that different host-types can represent distinct morphological and biological features and therefore often provide distinctive microniches for bacteria. This is particularly true in jellyfish where a life cycle can comprise such drastic transformations of the animal’s body plan that different life stages were initially thought to be different and unrelated animals (Agassiz, 1857). From the host’s perspective on the other hand, different life stages are associated with distinct host necessities, and these shifting host requirements likely help drive corresponding shifts in the structures of associated bacterial communities.

### Polyps as Microbial Reservoirs for Consecutive Life-Stages

While principal coordinates analysis and hierarchical clustering both demonstrated some overlap between the three investigated cyst types, polyps strongly separated out from cysts representing significantly different bacterial communities (**Figure 3** and **Table 1**). These results, again, are in agreement with findings from the jellyfish *A. aurita*, where polyps hosted significantly different bacterial communities from other life stages (Weiland-Bräuer et al., 2015). Weiland-Bräuer et al. (2015) hypothesized that a polyp-specific microbiota might be essential for their sessile lifestyle and possibly important for the initiation of later developmental stages. Taking into account that the polyp can be the base for a plethora of asexual reproductive strategies (reviewed in Adler and Jarms, 2009), it likely represents a reservoir of microbial members essential to the initiation, development, and survival of the subsequent life forms that derive from polyps. Aligned with this idea, we found that a much larger proportion of significantly differentially abundant OTUs were less abundant in polyps than in cysts (56 as opposed to only 7 OTUs which were more abundant in polyps than in cysts; Supplementary Table S4). Among those lower in abundance in polyps were members with closest relatives involved in chemolithoautotrophy, nitrogen fixation, and nitrogen and sulfur reduction (Supplementary Table S4) – functions that may have more of an environmental niche to fill in cyst life stages.

### Excysts – The Transition From Podocysts to Polyps

Polyps, podocysts, and excysts generally share a habitat but can survive under different conditions (Ishii et al., 2008; Levin et al., 2009; Thein et al., 2012). Looking at microbial similarities between life stages, polyps were more similar to excysts than to the two podocyst groups (Supplementary Figure S2 and **Table 1**). As excysts develop into polyps they share the same body plan and only differ in size, number of tentacles, food regime, and reproductive activity. Early excysts feed on nutrients provided by their podocyst capsule, and as they proceed growing more tentacles they increasingly feed on plankton, like their polyp counter-parts. Excysts are not known to reproduce until reaching the fully grown 16-tentacle stage; since the excysts in our study were sampled 2 days after hatching, on a developmental time scale they were much closer to the stage of a podocyst than that of a fully grown polyp (which is reached within ∼4 weeks). Hence, the close similarity between microbial communities of the excysts with those of the podocysts, while overall the excyst communities were found to be more similar to the polyps than the podocysts were, nicely demonstrates an early stage in the successive re-structuring of microbial communities from a podocyst to a polyp (**Figure 3** and Supplementary Figure S2).

### A Podocyst-Specific Microbiome

The survival of podocysts plays an important role in the abundance of jellyfish populations, and previous studies have speculated that the thick outer cuticle of podocyst capsules might act as a protective physical barrier to exclude microbes (Ikeda et al., 2011; Thein et al., 2013). However, we argue that such capsules are not only formed to protect the podocyst from the outside, but also to conserve a specific beneficial assemblage of microorganisms within the capsule. Compared to polyps and excysts, podocysts represent compact, encapsulated, dormant life stages with marginal metabolic activity. While these structures are not fully cryptobiotic, the scarcity of mitochondria, rough endoplasmic reticulum, and Golgi complexes in their cytoplasm, and a weak staining reaction for RNA, suggests only low metabolic activity (Ikeda et al., 2011). With these characteristics in mind, and considering that podocysts can survive for years, it is feasible that a specific microbiota helps to sustain the viability of podocysts – and potentially facilitates the activation of excystment. Identifying OTUs that were significantly more abundant in podocysts as compared to polyps, but not significantly more abundant in excysts as compared to polyps, resulted in 26 OTUs that may represent microbial members potentially important to the podocyst life stage including members of Bacteroidetes, Planctomycetes, Alpha-, Beta-, and Gammaproteobacteria, and Chloroflexi (Supplementary Table S6). An early study on *C. quinquecirrha* determined that podocysts used a significant proportion of their available protein during the period of encystment, and that the rate of reserve nutrients used was an order of magnitude lower than would be predicted from the measured rate of oxygen uptake – which raised the question of the fate of metabolic products in the podocyst, and whether soluble nitrogen compounds were accumulated or lost (Black, 1981). It would be particularly interesting to investigate in detail whether the podocyst microbiome is involved in such processes, and how.

A study of the marine dinoflagellate *Scrippsiella trochoidea*, reported the uptake of phosphorus by resting cysts and furthermore suggested that *Scrippsiella* cysts may be capable of N uptake; the question whether cyst associated bacteria may be involved in the process was raised (Rengefors et al., 1996). The acquisition of nutrients during the resting state in sediments was suggested to allow cysts to take advantage of high-nutrient environments and to maximize the survival of newly germinated cells. This could be equally possible for jellyfish podocysts.

The close proximity (tentacle-reach) of podocysts and mother polyps may have intuitively suggested that they would harbor microbial communities more similar to each other than polyps to the spatially segregated “isolated podocysts” would. However, at the level of resolution interrogated in the current study, (i.e., 97% OTUs), this idea was not statistically supported.

### The Core Microbiota of *C. plocamia*

The core microbiota of a host population can be defined as the fraction of microorganisms shared between animals of a studied species, independent of time, space, or life stage. Comparing the investigated life stages in *C. plocamia* revealed a stable microbiome consisting of 37 OTUs, with most of its members also found to be associated with other marine organisms (Supplementary Table S5). The closest known relatives of those consistently found to be present in *C. plocamia* are within clades known to be drivers of major elemental cycles, e.g., carbon (including methanotrophy), nitrogen, sulfur, and phosphorus (Supplementary Table S5). With best BLAST hits of these OTUs being from other marine, host-associated microbiomes such as sponges, sea squirts, and sea horses (Supplementary Table S5), the lineages of these taxa appear to have radiated outward with the diversity of animal life, and their conservation across these organisms suggests they may play fundamental roles in macroorganismal survivability. Considering the environmental characteristics of the Humboldt Current System, including the presence of different water masses, an extended oxygen minimum zone, and the existence of methane seeps (Sellanes et al., 2010), a functionally diverse core microbiome may facilitate the abundance of the jellyfish *C. plocamia* in this habitat.

### Microbial Members Consistently Present in *C. plocamia* and Also Found in *A. aurita*

Interestingly, 16 of the 37 OTUs consistently present in *C. plocamia* were also found to be present in *A. aurita* life-stages (Weiland-Bräuer et al., 2015). Organisms represented by these OTUs make interesting targets for further studies as they may play essential roles in scyphozoan jellyfish (**Table 2**). Differences in microbial member representation between the two jellyfish species most often occurred as present in *C. plocamia*, but absent in the *A. aurita* dataset. It is possible that the years of polyp-culture maintenance in artificial seawater employed in the Weiland-Bräuer study may have impeded the replenishment of, and/or possibly caused a loss of, some bacteria over time from the *A. aurita* microbiome. In contrast, in the current study, *C. plocamia* polyps were cultured under near-natural conditions in ambient seawater; such “real-world-conditions” may require a higher elasticity in the core microbial community composition of the host. There are also technical discrepancies to be considered, however. Namely, a greater sequencing depth was achieved in the current study, possibly allowing better recovery of representative sequences in *C. plocamia*. Also, different primer sets were utilized in the two studies, which may have resulted in varying effects of primer bias. Accordingly, the conserved presence of specific OTUs across these two jellyfish species likely carries more weight than any observed absence of an individual OTU does. The microbial members that are consistently present and shared across all investigated life stages of *C. plocamia*, as well as present in the microbiome of *A. aurita*, are of particular interest as they may have been essential to jellyfish for some time.

### Elemental Cycling Potential Within *C. plocamia* and *A. aurita*

Operational taxonomic units most closely related to a suite of metabolically and physiologically diverse microorganisms capable of mediating the interrelated pathways of carbon, nitrogen, sulfur, and phosphorus were identified in both *C. plocamia* and *A. aurita* (**Table 2**). Such microbial partners are likely to provide the host with a wide range of otherwise unavailable nutrients and other benefits. The deep-sea glass sponge *Lophophysema eversa*, for example, lives in close association with ammonia-, nitrite- and sulfur-oxidizing microbes that play essential roles as scavengers of toxic ammonia, nitrite and sulfide in the host (Tian et al., 2016).

Members most closely related to those known to be involved with carbon fixation, methanotrophy, and polycyclic aromatic hydrocarbon (PAH) degradation were also detected as part of the jellyfish microbiome. Methane is an energy source only available to methanotrophic microorganisms (Petersen and Dubilier, 2009), however, by associating with bacteria that can use one-carbon compounds, such as methanol or methane, the animal host is indirectly provided with nutrition from this source. Methanotrophic symbioses between marine invertebrates and bacteria have been reported in sponges (*Cladorhiza methanophila*), tubeworms (*Siboglinum* sp.), hydrothermal vent snails (*Ifremeria nautilei* and *Alviniconcha hessleri*), and deep-sea bathymodiolin mussels (*Bathymodiolus* and *Idas*), (reviewed in Petersen and Dubilier, 2009).

Polycyclic aromatic hydrocarbon are ubiquitous environmental pollutants and while most derive from anthropogenic activities, some PAHs occur naturally (Abdel-Shafy and Mansour, 2016) and originated from meteoric impacts in early Earth history. As such, and given the jellyfish’s deep evolutionary roots, jellyfish-associated PAH-degraders might represent ancient microbial-animal associations. A recent study (Almeda et al., 2013) revealed a high tolerance of *A. aurita* medusae to crude oil exposure and highlighted their ability to bio-accumulate PAHs. This may be partially attributable to PAH-degrading microbial associations and may facilitate the capacity of jellyfish to inhabit and increase in abundance in polluted coastal habitats. Studies monitoring the microbial communities of jellyfish before, during, and after exposure to crude oil could shed light on this.

**FIGURE 5:**
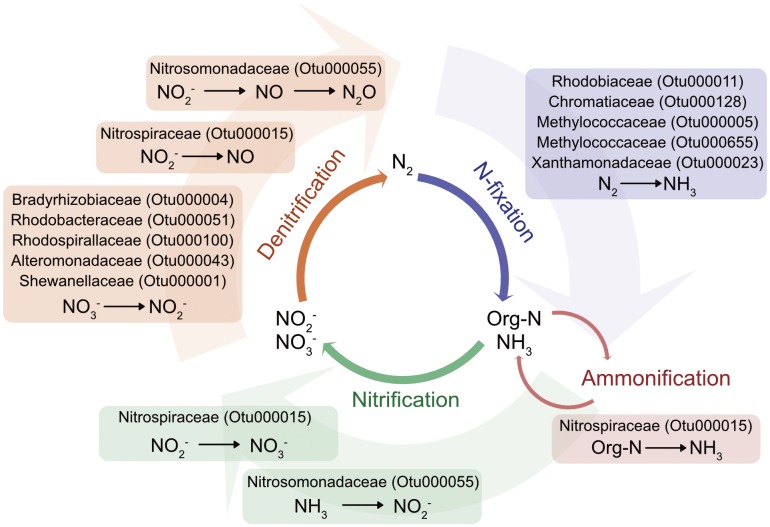
Schematic of nitrogen-cycling potential of microbial members identified as part of *C. plocamia*’s core microbiome. (More detailed information about each OTU is presented in **Table 2** and Supplementary Table S7).

Nitrogen is vital to the structures and biochemical processes defining life (Francis et al., 2007), and even though it is the most abundant element in the atmosphere, this di-nitrogen molecular form is not bioavailable. As such it is often limiting in coastal and open-ocean marine ecosystems and needs to be converted into ammonium by nitrogen-fixing microbes before being readily available to other organisms. Ammonium is then either recycled in the water column or is oxidized to nitrite and nitrate. Further processes, including denitrification and anammox return nitrogen back to the atmosphere (Lyons et al., 2015). Symbiotic nitrogen fixers are known to be associated with a variety of marine invertebrates such as wood-boring bivalves (Lechene et al., 2007), corals, sponges, and sea urchins (reviewed in Fiore et al., 2010). Adding to this list, the jellyfish microbiome encompasses a suite of bacteria whose nearest relatives have the capacity to transform the element through an almost complete nitrogen cycle (i.e., nitrogen fixation, nitrification, ammonification, and denitrification; **Table 2** and **Figure 5**).

Nitrifying bacteria are also known to harbor key enzymes involved in the conversion of ammonia to hydroxylamine, and hydroxylamine to nitric oxide (NO), an important messenger molecule known to regulate metamorphosis in marine invertebrates (Bishop and Brandhorst, 2003), to regulate swimming in the jellyfish *Aglantha digitale* (Moroz et al., 2004), and to facilitate the discharge of nematocytes in the small sea anemone *Aiptasia diaphana* (Salleo et al., 1996). Considering the important roles NO plays in cnidarians, symbiotic partnerships with bacteria providing this indispensable molecule could be essential.

The sulfur cycle is tightly linked to the carbon cycle and includes reductive and oxidative processes. In some coastal marine sediments more than 50% of microbiologically degraded organic matter occurs via sulfate reduction (Lyons et al., 2015). Sulfur-oxidizing and sulfate-reducing bacteria were identified as part of the jellyfish microbiome. The bivalve *Codakia orbicularis* is known to live in symbiotic association with a sulfur-oxidizing bacterium that not only sustains the bivalve’s carbon demands through sulfide oxidation, but also supplies fixed nitrogen (König et al., 2016).

Alkaline phosphatase (AP) is an enzyme found in many organisms that is involved in dephosphorylation processes of organic matter in the eutrophic marine environment where dissolved DNA, RNA, and proteins are thought to be the main sources of organic phosphorus for bacteria and phytoplankton (Gonzales De Canales and Martin Del Rio, 1985; Paul et al., 1990). Moreover, the mussel *Crenomytilus grayanus* lives in symbiosis with the bacterial strain *Cobetia marina* that produces highly active AP and has been suggested to play an active role in the process of shell formation in its host (Plisova et al., 2005). AP has also been found in the jellyfish *C. quinquecirrha* (Stewart and Lakshmanan, 1975) and sequences most closely related to AP-positive bacteria have been detected in the jellyfish microbiome in the current study.

## Conclusion

This study identified potentially ubiquitous bacterial members across scyphozoans from two families. The conservation of such members implies functional roles that may be integral to the host’s proliferation, and can reveal insights into the co-evolution of one of the earliest animals and its microbiome. We can now target jellyfish microbiomes with metagenomic/metatranscriptomic approaches to begin deciphering what exactly these roles may be, and how they may be involved in the evolutionary success of scyphozoan jellyfish.

## Data Accessibility

The raw Illumina MiSeq sequence data generated herein have been uploaded to NCBI’s Sequence Read Archive (SRA) under BioProject accession number PRJNA413177.

## Ethics Statement

This study was approved by the Comité de Ética de Investigación Científica, University of Antofagasta.

## Author Contributions

JC designed the study and carried out the field- and lab work. ML and JK analyzed the data. RA contributed to the field- and lab work, and provided lab space and reagents. JC and ML drafted the manuscript. All authors provided comments and approved the final manuscript.

## Conflict of Interest Statement

The authors declare that the research was conducted in the absence of any commercial or financial relationships that could be construed as a potential conflict of interest.
