# Supplementary material for: Jellyfish Life Stages Shape Associated Microbial Communities, While a Core Microbiome Is Maintained Across All
Source: Front Microbiol. 2018 Jul 12;9:1534. doi: 10.3389/fmicb.2018.01534 (PMC6052147; doi:10.3389/fmicb.2018.01534)
Supplement: Supplementary file 2 [file Data_Sheet_2.DOCX]

Figure S1: Distribution of proportions of reads recovered across each group by major taxa. N=4 for each life stage. Number in parentheses following taxon name indicate the number of OTUs found within that taxon. Error bars plotted are standard errors. P-values indicate results from ANOVA. The only ANOVA found to have a p-value < 0.05 was within the Betaproteobacteria, to which Tukey’s Honest Significant Difference test revealed a difference between the polyps and isolated cyst groups.

Figure S2: Hierarchical clustering of mean within- and between-group Bray-Curtis dissimilarities. Dendrogram tips hang to within-group mean dissimilarities. Groups are fused into clusters at their respective between-group dissimilarities.
